# Effect of two contemporary root canal sealers on root canal dentin microhardness

**DOI:** 10.4317/jced.53052

**Published:** 2017-01-01

**Authors:** Maram E. Khallaf

**Affiliations:** 1 Researcher at Restorative and Dental Materials Research department, National Research centre, Giza. Egypt

## Abstract

**Background:**

Successful root canal treatment depends on proper cleaning, disinfecting and shaping of the root canal space. Pulpless teeth have lower dentin microhardness value compared to that of vital teeth. A material which can cause change in dentin composition may affect the microhardness. Thus the aim of this study was to evaluate and compare the effect of two root canal sealers on dentin microhardness.

**Material and Methods:**

Forty two single rooted teeth were selected and divided into 3 equal groups; Apexit, iRootSP and control groups (n=14) Each group was then divided into 2 subgroups according to the post evaluation period; 1 week and 2 months (n=7). Root canal procedure was done in the experimental groups and obturation was made using either; Apexit, iRootSP or left unprepared and unobturated in the control group. Roots were sectioned transversely into cervical, middle and apical segments. The three sections of each root were mounted in a plastic chuck with acrylic resin. The coronal dentin surfaces of the root segments werepolished. Microhardness of each section was measured at 500 µm and 1000 µm from the canal lumen.

**Results:**

Four way-ANOVA revealed that different tested sealer materials, canal third, measuring distance from the pulp and time as independent variables had statistically non significant effect on mean microhardness values (VHN) at *p*≤0.001. Among iRootSP groups there was a statistically significant difference between iRoot SP at coronal root portion (87.79±17.83) and iRoot SP at apical root portion (76.26±9.33) groups where (*p*=0.01). IRoot SP at coronal canal third had higher statistically significant mean microhardness value (87.79±17.83) compared to Apexit at coronal third (73.61±13.47) where (*p*=0.01).

**Conclusions:**

Root canal sealers do not affect dentin microhardness.

** Key words:**Root canal, dentin, sealers, microhardness, bioceramic.

## Introduction

Successful root canal treatment depends on proper cleaning, disinfecting and shaping of the root canal space, followed by proper obturation. Root canal treatment has been generally correlated with decrease in tooth strength. During the process of root canal treatment dentin microhardness is one of the strength properties which are changed. This might be due to compositional changes linked with caries process, pulpectomy and the application of restorative materials (1-3).

It was shown that pulpless teeth have lower dentin microhardness value compared to that of vital teeth (4). Also, the biomechanical properties of dentin are changed after the loss of tooth vitality (5,6). Since the composition and surface changes of the tooth structure affect dentin microhardness (7), the effect of different chemicals such as bleaching agents, chloroform and chelating agents on dentin hardness were previously studied (8-10). Also the effect of endodontic irrigation solutions on microhardness of root canal dentin was evaluated (11).

Calcium and phosphorus present in hydroxyapatite crystals are the main inorganic composition of dental hard tissue. Measuring dentin microhardness, considered as an indirect indication for losing or gaining of mineral content in the dental hard tissues. Thus a material which can cause change in dentin composition may affect the microhardness, in addition to the permeability and solubility of the root canal dentin (11).

Thus the aim of this study was to evaluate the effect of two contemporary root canal sealers on the root canal dentin microhardness.

## Material and Methods

-Teeth selection and preparation

A study protocol has been approved by the ethical committee at the National Research Centre, Egypt. Forty two freshly extracted single rooted teeth were used in this study. After removal of calculus and soft-tissue debris, the teeth were stored at 4°C in tap water. Working length was adjusted at 22 mm. Fourteen teeth were left unprepared and unobturated to serve as negative control. Access cavity was prepared in the remaining 28 teeth, root canal preparation was done using protaper universal system till size F3 using 5ml 5%NaOCl irrigation after each file. Following this procedure, irrigation with 2 mL of 17% EDTA solution for 3 min was performed. A final rinse was performed with distilled water.

-Obturation procedure

The prepared roots were then divided into two groups (G); according to the sealer used for obturation (n=14) G1: Apexit sealer (Ivoclar, Viva Dent), G2: iRoot SP sealer (Innovative Bioceramix, Vancouver, Canada).Each sealer was mixed according to manufacturer’s instructions. Obturation was done using Protaper Universal Guttapercha. Excess Guttapercha was seared off and condensed 1 mm below the canal orifice. Teeth in the 2 experimental groups and the control group were randomly divided into equal subgroups (n=7) according to the evaluation periods either; 1 week or 2 months. Restored teeth were stored in distilled water at 37ºC for the evaluation periods.

-Specimen preparation for microhardness evaluation

After each evaluation period the roots were transversely sectioned under copious water spray into three equal sections; cervical, middle and apical sections using a double sided diamond disc. The three sections of each root were then mounted horizontally, apart from each other, in a metal chuck with auto-curing acrylic resin material. The embedded dentin specimens were then polished gradually with a carborundum paper disc, up to 1200 grade under running water followed by a final polish using 0.1 µm of diamond paste.

-Microhardness measurment

The microhardness measurements were performed by using a Vickers Diamond Microhardness Tester (Nexus 4000/60, INNO-VATEST, Netherlands, Europe) in Vickers Hardness Units (VHN).The microhardness measurements were taken at three different points at each root third, at depths of 500 µm and 1000 µm from the lumen. Each measurement was carried out by using a 300g load for 20 second Dwell time.

-Statistical analysis

The mean and standard deviation of microhardness values were calculated for each group. Data were explored for normality using Kolmogorov-Smirnov and Shapiro-Wilk tests and showed parametric (normal) distribution. Applying ANOVA followed by Tukey’s post-hoc test to compare between different variables. The significance level was set at *P* ≤ 0.05. Statistical analysis was performed with IBM® SPSS® Statistics Version 20 for Windows.

## Results

Four way-ANOVA revealed that different tested sealer materials, canal third, depth from lumen and time as independent variables had statistically non significant effect on mean microhardness values (VHN) at *p*≤0.001. Where there was no statistically significant difference between (iRoot SP) (80.41±15.45), (Apexit) (77.18±17.41) and (Control) (78.83±14.60) where (*p*>0.05). Also there was no statistically significant difference between 1 week (80.06±15.49) and 2 months (77.53±17.43) where (*p*=0.3).

There was no statistically significant difference between coronal third (80.70±17.17) on one side and each of middle third (80.77±19.26) and apical third (74.92±11.78) on the other side where (*p*=0.9) and (*p*=0.05) respectively. Also no statistically significant difference was found between middle (80.77±19.26) and apical thirds (74.92±11.78) where (*p*=0.09).

Moreover there was no statistically significant difference between the hardness at different distances from the pulp (500µm) (79.72±18.39) and (1000µm) (77.87±14.40) where (*p*=0.4). A statistically significant difference was found between (iRoot) at coronal third (87.79±17.83) and (Apexit) at the coronal third (73.61±13.47) where (*p*=0.01).

Mean and standard deviation of the root canal dentin microhardness of the two tested sealers (Apexit and iRoot SP) and Canal Third (Coronal, Middle and Apical), time (1 Week and 2 Months) and distance from pulp (500 µm) are presented in  Table 1.

**Table 1:**
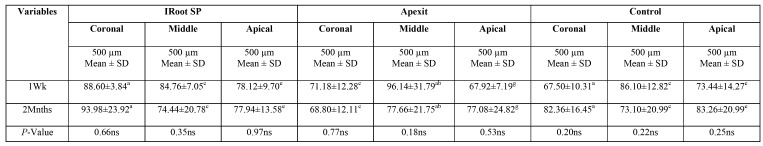
Mean and standard deviation of the root canal dentin microhardness of the two tested sealers (Apexit and iRoot SP) and Canal Third (Coronal, Middle and Apical), time (1 Week and 2 Months) and distance from pulp (500 µm).

Mean and standard deviation of the root canal dentin microhardness of the two tested sealers (Apexit and I-root SP) and canal third (Coronal, Middle and Apical), time (1 Week and 2 Months) and distance from pulp (1000 µm) are presented in  Table 2.

**Table 2:**
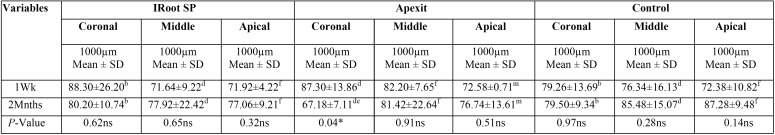
Mean and standard deviation of the root canal dentin microhardness of the two tested sealers (Apexit and iRoot SP) and Canal Third (Coronal, Middle and Apical), time (1 Week and 2 Months) and distance from pulp (1000 µm).

## Discussion

Hardness measurements are commonly used to obtain an indication of the mineral content of the hard tissue of teeth (12). Microhardness measurement was tested at the coronal, middle and apical thirds of the root canal dentin as the tubular density varies from an area to another on the root dentin surface and this can affect dentin microhardness (13).

Vickers microhardness tester was selected over Knoop hardness tester in this study, as Vickers test is more appropriate for evaluating surface changes of deeper dental hard tissues (14).

In this study, standardization of the specimens’ instrumentation, irrigation and obturation procedure was done which is an important factor for accurate results.

IRoot SP is a bioceramic sealer which hardens only when exposed to fluids in the dentinal tubules. The hydration reaction produces hydroxyapatite which forms a chemical bond with dentinal wall (15).

Apexit is a calcium hydroxide based sealer which produces calcium hydroxide upon setting. Also the bioceramic based sealers contain calcium hydroxide in addition to its ability to form hydroxyapatite which forms a bond between sealer and dentinal wall (16).

Results showed that there was no statistically significant difference of the mean dentin microhardness between groups of the tested sealers (iRoot SP and Apexit) and control group at both evaluation time, and measuring distance. Although it has been said that root canal treatment weakens the root dentin (17), results of this study showed that both sealers didn’t significantly affect dentin microhardness when compared to control group. This can be attributed to the use of sealers which could increase dentin strength as shown by Ghoneim, *et al.* (15) where the use of a bioceramic sealer proved to increase resistance to fracture. The fact that microhardness was not affected after obturation can be attributed to the use of sealers that produce calcium hydroxide and hydroxyapatite that diffuse into dentinal tubules (16,18).

It was also shown that regarding the bioceramic sealer (iRoot SP) there was a statistically significant difference between micro-hardness at coronal portion and that at apical portion. This can be explained by the ability of bioceramic sealers to form hydroxyapatite to bond with dentin. Bonding with dentin is affected by the number of exposed dentinal tubules available for bonding. Use of irrigating solution is a requirement for efficient removal of the smear layer and pulp debris which in turn affect sealing ability of filling materials. NaOCl used in this study was reported to be ineffective irrigant to remove both the organic and inorga-nic components of the smear layer (19-21). Its physicochemical action is restricted to the organic particles. Thus NaOCl coupled with EDTA can remove the inorganic debris formed in the instrumented root canals, mainly in the middle and cervical thirds (22). Many studies reported that the efficacy of EDTA to remove the smear layer decreased from the coronal third to apical third of the root (23). This can be explained as the flow and backflow of the fluid are reduced in the apical third (24). While more abundant and larger dentinal tubules coronally (25) exposes the dentin to a high amount of irrigants and allow for a better flow of the solution thus enhancing the effectiveness of smear layer removal (26-28) which leads to more bonding with the produced hydroxyapatite. This increased deposition of hydroxyapatite at the coronal level can also explain the obtained result that regardless of time VHN for iRoot SP at the coronal section was significantly higher than that of Apexit.

It was also found in this study that there was no reduction in microhardness as the pulp is apporoached at 500 µm in comparison to 1000 µm this is in contrary to findings of Slutzky-Goldberg, *et al.* (29) who stated that microhardness decrease as we approach the pulp, but the difference in the results could be due to measuring of microhardness before obturation in that study while in this study dentin microhardness closer to the pulp is more affected by calcium hydroxide which infiltrates the dentinal tubules.

## Conclusions

Root canal treatment and sealers do not affect radicular dentin microhardness.
